# Lipopolysaccharides from *Microcystis* Cyanobacteria-Dominated Water Bloom and from Laboratory Cultures Trigger Human Immune Innate Response

**DOI:** 10.3390/toxins11040218

**Published:** 2019-04-11

**Authors:** Zdena Moosová, Lenka Šindlerová, Barbora Ambrůzová, Gabriela Ambrožová, Ondřej Vašíček, Mirna Velki, Pavel Babica, Lukáš Kubala

**Affiliations:** 1 Department of Biophysics of Immune System, Institute of Biophysics, Academy of Sciences of the Czech Republic, 61265 Brno, Czech Republic; moosova@recetox.muni.cz (Z.M.); sindler@ibp.cz (L.Š.); b.ambruzova@gmail.com (B.A.); ambrozova@ibp.cz (G.A.); ondrej.vasicek@ibp.cz (O.V.); 2 RECETOX, Faculty of Science, Masaryk University, 62500 Brno, Czech Republic; pavel.babica@gmail.com; 3 Institute of Experimental Biology, Faculty of Science, Masaryk University, 62500 Brno, Czech Republic; 4 Department of Biology, Josip Juraj Strossmayer University of Osijek, 31000 Osijek, Croatia; mvelki@biologija.unios.hr

**Keywords:** water bloom, cyanobacteria, endotoxin, lipopolysaccharide, inflammation, leukocytes

## Abstract

Massive toxic blooms of cyanobacteria represent a major threat to water supplies worldwide. Here, the biological activities of lipopolysaccharide (LPS) isolated from *Microcystis aeruginosa*, the most prominent cyanobacteria in water bloom, were studied. LPS was isolated from complex environmental water bloom samples dominated by *M. aeruginosa*, and from laboratory cultures of non-axenic as well as axenic *M. aeruginosa* strains PCC7806 and HAMBI/UHCC130. Employing human blood-based in vitro tests, the LPS isolated from complex water bloom revealed the priming of both major blood phagocyte population monocytes and polymorphonuclear leukocytes documented by the increased surface expression of CD11b and CD66b. This was accompanied by a water bloom LPS-mediated dose-dependent induction of tumor necrosis factor α, interleukin-1β, and interleukin-6 production. In accordance with its priming effects, water bloom LPS induced significant activation of p38 and ERK1/2 kinases, as well as NF-κB phosphorylation, in isolated polymorphonuclear leukocytes. Interestingly, the pro-inflammatory potential of LPS from the axenic strain of *M. aeruginosa* was not lower compared to that of LPS isolated from non-axenic strains. In contrast to the biological activity, water bloom LPS revealed almost twice higher pyrogenicity levels compared to *Escherichia coli* LPS, as analyzed by the PyroGene test. Moreover, LPS from the non-axenic culture exhibited higher endotoxin activity in comparison to LPS from axenic strains. Taking the above findings together, *M. aeruginosa* LPS can contribute to the health risks associated with contamination by complex water bloom mass.

## 1. Introduction 

Cyanobacteria are photosynthetic gram-negative bacteria involving various genera, living in freshwater or estuarine planktonic habitats [1,2]. Massive growths of planktonic cyanobacteria in water reservoirs, leading to the formation of so-called cyanobacterial water blooms, have intensified over the last decades due to anthropogenic activities and become a fundamental environmental challenge [2]. Genus *Microcystis* represents one of the most common and pervasive cyanobacteria, whose blooms can be found in tropical and temperate regions across the world [1,2]. Environmental cyanobacterial water blooms are complex systems consisting primarily of cyanobacteria with different levels of contamination by other heterotrophic bacteria [3]. Cyanobacteria produce many different toxins with a wide spectrum of biological effects on humans, often very toxic, that represent a major threat to water supplies worldwide [2,4,5].

Up to now, most attention has been focused on the hepatotoxic and hepatocarcinogenic effects of cyanobacterial toxins, especially microcystins (considered to be one of the most toxic and abundant cyanotoxins) [6]. However, among the most important acute clinically-relevant pathologies associated with cyanobacterial blooms are acute inflammatory gastrointestinal diseases, with hemorrhage [7,8], nausea, vomiting [9,10], and abdominal pain [9] as the main clinical manifestations. At least a proportion of these health effects could be associated with exposure to cyanobacterial lipopolysaccharide (LPS), according to published observations of gastrointestinal (GIT), skin, and eye irritation, headaches, cramps, fever, and other effects summarized in Stewart et al. 2006 [11] and Durai et al. 2015 [12]. Interestingly, not only contaminated drinking water but also recreational exposure to cyanobacterial blooms through swimming [13,14,15,16], water-skiing [15], or canoeing [16] can result in adverse health effects, for example, in gastroenteritis.

However, there is very high heterogeneity among cyanobacterial metabolites that possess the ability to induce pathological effects in humans. Thus, mechanisms of the induction of acute and chronic adverse or toxic effects by cyanobacterial water blooms are currently not well understood. Most studies currently focus on secondary metabolites, such as microcystin-LR [17,18]. Interestingly, only limited knowledge is available regarding cyanobacterial water bloom lipopolysaccharides (LPS), also known as endotoxins. LPS is the main component of the gram-negative bacterial cell wall, which can trigger a complex systemic inflammatory response. However, the biological potential of LPS varies greatly due to structural differences among individual gram-negative bacteria species and even strains [4,11,12,19,20]. *M. aeruginosa* LPS was reported to contain a large quantity of keto-deoxyoctulosonate, 3-deoxy sugars, glucosamine, fatty acids, fatty acid esters, hexoses, and phosphates, also present in LPS structures of other gram-negative bacteria [4]. Nevertheless, data regarding the structure of *M. aeruginosa* LPS varies, since other studies failed to find the keto-deoxyoctulosonate domain and heptose, but, on the other hand, identified the presence of specific oxidized fatty acids and rare types of sugars [21,22,23]. Thus, to speculate on the biological effects of *M. aeruginosa* LPS or LPS from cyanobacteria in general on the basis of its structure alone would be questionable [2].

LPS present in an environment can be recognized and can activate different types of cells in the body, including keratinocytes and lining epithelia of the gastrointestinal tract, airways, and lungs [11]. However, the most potent pathological effects are from LPS that enters systemic blood circulation through the altered intestinal barrier. The increased permeability of intestinal epithelium is reported to be related to numerous pathological conditions with the symptoms of gastroenteritis [24]. LPS entering systemic circulation can induce the chronic or even acute systemic inflammatory response of the organism known as endotoxemia [25,26]. Bacterial LPS is principally recognized by innate immune cells that are among the most sensitive to LPS and recognize LPS by its surface receptors, primarily the complex of Toll-like receptor 4 (TLR4), CD14, and MD2 [27]. Activation of these receptors leads to a multifaceted cell signaling response, involving especially p38 and ERK1/2 mitogen-activated protein kinases (MAPK) and nuclear factor-κB (NF-κB) transcription factor [27]. In humans, the innate immune cells in systemic circulation are represented by monocytes and neutrophil granulocytes (polymorphonuclear leukocytes (PMNLs)), which are the most abundant phagocytes in blood. These phagocytes are primed by LPS leading to an increase in the surface expression of their receptors (e.g., CD11b, CD66) through the degranulation of specific granules [28]. Further, the intensive production of different pro-inflammatory cytokines occurs, particularly tumor necrosis factor α (TNF-α), interleukin 6 (IL-6), IL-8, and IL-1β, that are responsible for systemic response [29]. However, the potential of LPS to activate cells depends on the potential of a particular LPS structure to be recognized and to trigger receptor activation [4,11,12,19,20]. Thus, the pathological severity of the organism response to LPS relates to the potential of particular LPS to activate cells and to the number of LPS entering the systemic circulation.

The focus of the present study was to assess the potential of LPS from the cyanobacteria *M. aeruginosa* to activate human blood phagocytes. An environmental sample of water bloom dominated by *M. aeruginosa* was used to isolate LPS. Such LPS represents an environmentally relevant mixture of LPS from the dominant cyanobacterium, as well as from bloom-associated gram-negative heterotrophic bacteria. In order to evaluate the biological activity of *Microcystis* LPS alone, the effects of LPS isolated from unialgal *Microcystis* laboratory cultures were also studied. However, many established laboratory strains of cyanobacteria are non-axenic; that is, they also contain heterotrophic bacteria [30,31]. Although toxicity, as well as ecotoxicity, of LPS isolated from different cultured cyanobacteria, have been investigated [32,33,34,35,36,37], in most of the studies, the issue of axenicity of cyanobacterial cultures used for LPS isolation and the possible contribution of LPS from heterotrophic bacteria was not specifically addressed. Therefore, we decided to assess the activity of LPS from both types of *Microcystis* cultures, that is, non-axenic (closer to environmental conditions with the presence of heterotrophic bacteria) and axenic (purely cyanobacterial) *M. aeruginosa* PCC7806. Further, to capture the strain specificity of LPS biological activity, another axenic strain, *M. aeruginosa* HAMBI/UHCC 130, was used.

## 2. Results

### 2.1. LPS Isolated from M. aeruginosa Water Bloom Induces the Production of Pro-Inflammatory Cytokines

**Figure 1:**
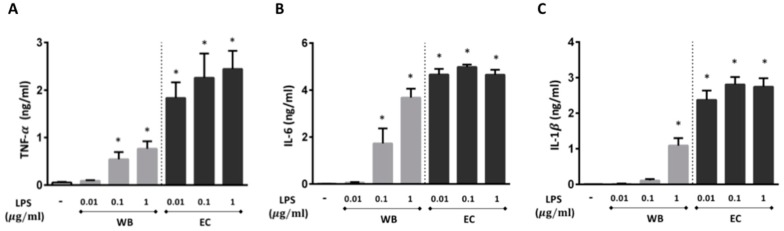
Potential of lipopolysaccharide (LPS) isolated from an environmental water bloom sample dominated by *M. aeruginosa* (WB) to induce TNF-α (**A**), IL-6 (**B**), and IL-1β (**C**) production in whole blood. Blood was treated with WB in the concentration range from 0.01 to 1 µg/ml for 24 h at 37 °C. *E. coli* LPS (EC) was used as a positive control in the same concentration range. Data are presented as mean +/− SEM (*n* = 5). Statistical significance was determined by one-way analysis of variance ANOVA, followed by Dunnett’s test (* *p* < 0.05).

The potential of LPS isolated from *M. aeruginosa*-dominated water bloom (labeled WB throughout the text) to initiate production of pro-inflammatory cytokines was documented by statistically significant increase in the production of TNF-α and IL-6 at concentrations of 0.1 and 1 µg/mL (Figure 1A,B) and of IL-1β at 1 µg/mL (Figure 1C) after the treatment of human whole blood for 24 h at 37 °C. A robust increase of these cytokines was also induced by LPS from *E. coli* (labeled EC throughout the text) that was used as a positive control (Figure 1).

### 2.2. LPS Isolated from M. aeruginosa Water Bloom Activates Blood Phagocytes

**Figure 2:**
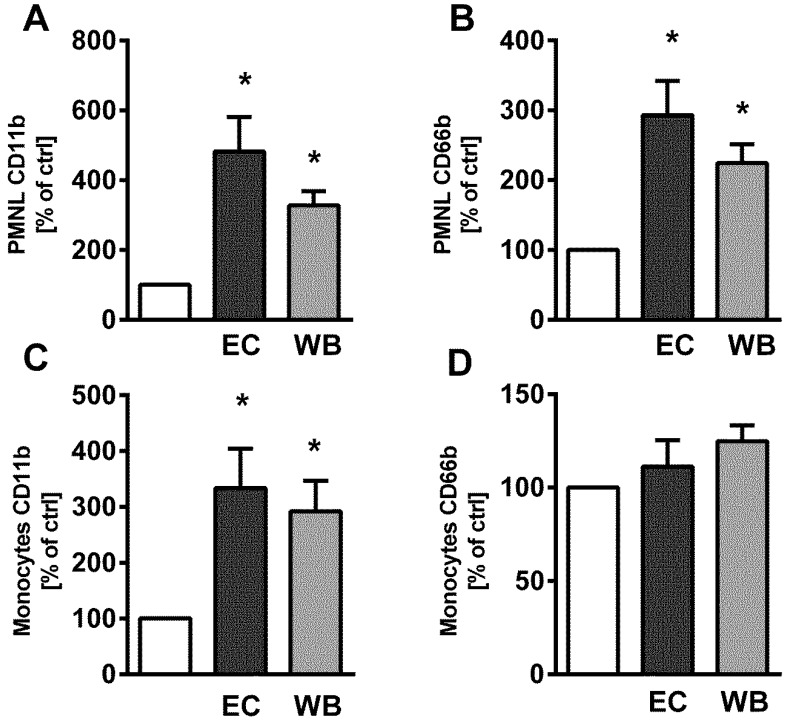
Surface expression of CD11b (**A**,**C**) and CD66b (**B**,**D**) on polymorphonuclear leukocytes (PMNLs) (CD14^low^) (**A**,**B**) and monocytes (CD14^high^) (**C**,**D**) induced by lipopolysaccharide (LPS) isolated from an environmental water bloom sample dominated by *M. aeruginosa* (WB) and LPS from *E. coli* (EC) (both 1 µg /mL) in whole blood after 6 h at 37 °C. Data are presented as the percentage of control untreated samples (mean +/− SEM; *n* = 5). Statistical significance was determined by one-way analysis of variance ANOVA, followed by Dunnett’s test (* *p* < 0.05).

The potential of WB to activate the two major blood phagocyte populations, PMNLs and monocytes, was determined according to the increase in the surface expression of CD11b and CD66b. WB (1 µg/mL) induced a significant increase in the surface expression of CD11b on both PMNLs (Figure 2A) and monocytes (Figure 2C) similar to control EC after the incubation of whole blood with LPS for 6 h. The surface expression of CD66b was also elevated (Figure 2B,D); however, statistical significance was reached only in the case of PMNLs (Figure 2D).

### 2.3. LPS Isolated from M. aeruginosa Water Bloom Activates Selected Signaling Pathways

**Figure 3:**
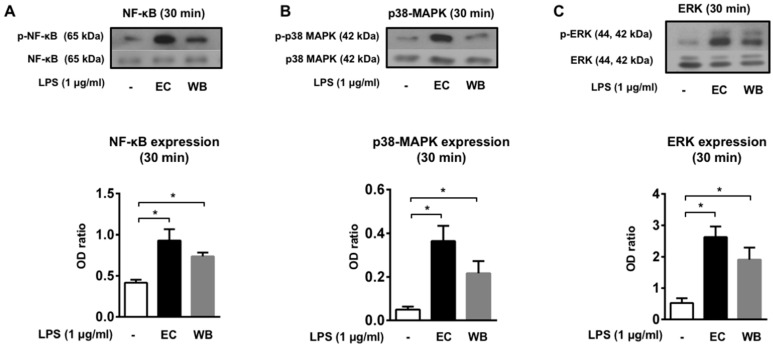
Phosphorylation of nuclear factor-κB (NF-κB) (**A**), p38 kinase (**B**), and extracellular signal-regulated kinase (ERK) 1/2 kinase (**C**) in human isolated polymorphonuclear leukocyte (PMNL) after 30 min of treatment with lipopolysaccharide (LPS) isolated from an environmental water bloom sample dominated by *M. aeruginosa* (WB) and LPS from *E. coli* (EC) (1 µg/mL). Levels of phosphorylated proteins and the total form of such proteins are shown on representative western blots. Data are presented as mean +/− SEM of the ratio of the optical density (OD) of phosphorylated form to the optical density of total protein (*n* = 3). Statistical significance was determined by one-way analysis of variance ANOVA, followed by Dunnett’s test (* *p* < 0.05).

The activation of selected signaling pathways, ERK1/2, NF-κB, and p38, was observed in isolated PMNLs treated with WB for 30 min (Figure 3). Interestingly, WB (1 µg/mL) induced a significant increase in the phosphorylation of transcriptional factor NF-κB (Figure 3A) and both kinases, p38 (Figure 3B) and ERK1/2 (Figure 3C). Similarly, the positive control EC (1 µg/mL) induced a significant increase in the phosphorylation of all studied proteins (Figure 3).

### 2.4. Cyanobacterial LPS Alone also Activates Blood Phagocytes

**Figure 4:**
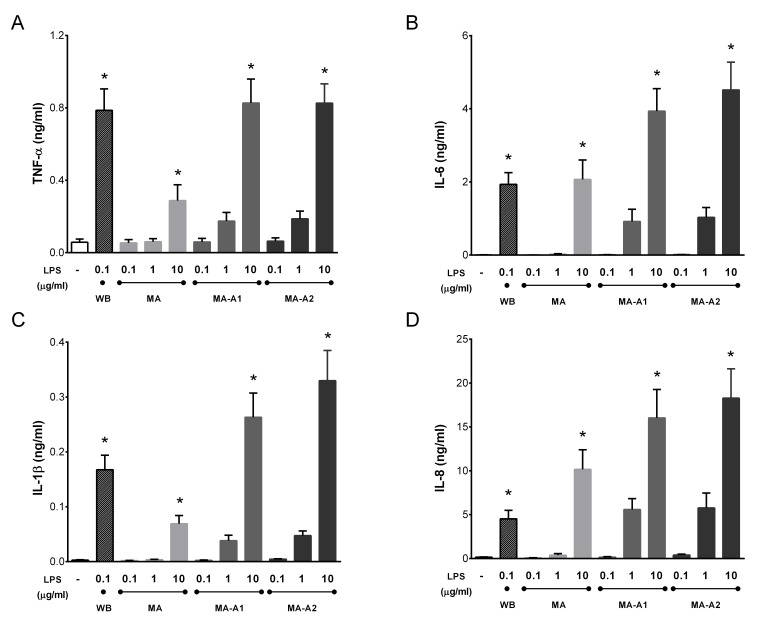
Induction of tumor necrosis factor α (TNF-α) (**A**), interleukin 6 (IL-6) (**B**), IL-1β (**C**), and IL-8 (**D**) in whole blood treated with lipopolysaccharide (LPS) isolated from the non-axenic *M. aeruginosa* strain (MA) and two axenic *M. aeruginosa* strains (MA-A1 and MA-A2) in the concentration range 0.1–10 µg/mL for 24 h at 37 °C. As the positive control, 0.1 µg/mL of LPS isolated from an environmental water bloom sample dominated by *M. aeruginosa* (WB) was used. Data are presented as mean +/− SEM (*n* = 5). Statistical significance was determined by one-way analysis of variance ANOVA, followed by Dunnett’s test (* *p* < 0.05).

The incubation of diluted whole blood with all *Microcystis* LPSs MA (non-axenic culture), MA-A1 (axenic *M. aeruginosa* PCC 7806), and MA-A2 (axenic *M. aeruginosa* HAMBI/UHCC130) caused a dose-dependent increase in the production of the four studied pro-inflammatory cytokines TNF-α (Figure 4A), IL-6 (Figure 4B), IL-1β (Figure 4C), and IL-8 (Figure 4D). The statistically significant effects were observed at the highest concentration (10 µg/mL) (Figure 4).

**Figure 5:**
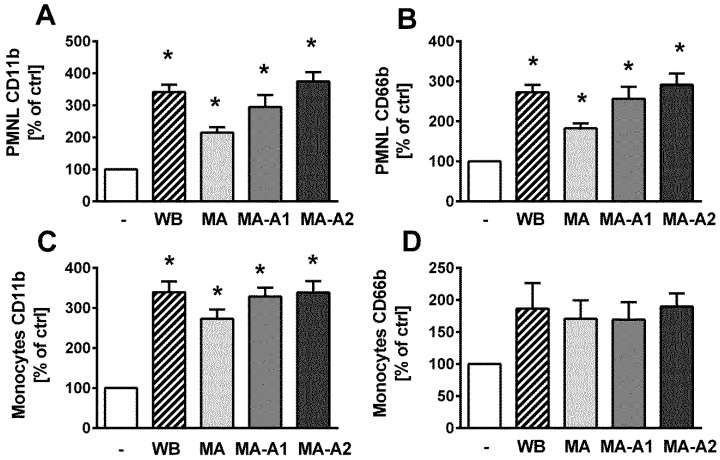
Surface expression of CD11b (**A**,**C**) and CD66b (**B**,**D**) on polymorphonuclear leukocytes (PMNLs) (CD14^low^) (**A**,**B**) and monocytes (CD14^high^) (**C**,**D**) induced by lipopolysaccharide (LPS) isolated from the non-axenic *M. aeruginosa* strain (MA) and two axenic *M. aeruginosa* strains (MA-A1 and MA-A2) at a concentration of 10 µg/mL in whole blood after 6 h at 37 °C. As the positive control, 0.1 µg/mL of LPS isolated from an environmental water bloom sample dominated by *M. aeruginosa* (WB) was used. Data are presented as the percentage of untreated control blood (mean +/− SEM; *n* = 5). Statistical significance was determined by one-way analysis of variance ANOVA, followed by Dunnett’s test (* *p* < 0.05).

Further, the significant pro-inflammatory potential of LPS isolated from both non-axenic and axenic *Microcystis* cultures was confirmed by testing the activation of the blood phagocytes PMNLs and monocytes according to the increase in the surface expression of CD11b and CD66b. In the case of PMNLs, all cyanobacterial LPSs caused a significant increase in the surface expression of CD11b (Figure 5A) and CD66b (Figure 5B) after whole blood exposure to LPSs (10 µg/mL) for 6 h. In the case of monocytes, a significant increase in the expression of CD11b was observed (Figure 5C), while the increase in CD66b expression did not reach statistical significance (Figure 5D).

### 2.5. Pyrogenic Activity of Tested LPSs

The pyrogenic activity of all tested LPS, determined by PyroGene test, is shown in Table 1. The highest pyrogenicity was detected in LPS isolated from the environmental sample of water bloom. Surprisingly, it was almost two times higher than the pyrogenicity of EC. The pyrogenicity of LPS isolated from *M. aeruginosa* cultures was much lower than the EC. The EC was approximately six times more pyrogenic than LPS from the non-axenic culture (MA), and approximately 700 (MA-A1) or 2000 (MA-A2) times more pyrogenic than LPS from axenic cultures.

## 3. Discussion

We report that LPS from water bloom dominated by *M. aeruginosa* revealed a significant potential to induce complex inflammatory responses in human blood associated with the activation of major phagocyte populations. Interestingly, LPS isolated from axenic and non-axenic strains of *M. aeruginosa* revealed comparable pro-inflammatory potential.

These results are in line with our previously published observation that LPS extracted from complex cyanobacterial water bloom has a substantial potential to induce significant inflammatory response accompanied by the production of pro-inflammatory cytokines by blood cells [32]. In the present study, we showed that both major populations of blood phagocytes, PMNLs and monocytes, were activated after exposure to LPS isolated from water bloom dominated by *M. aeruginosa*, as well as LPS isolated from axenic and non-axenic strains of *M. aeruginosa*. The activation of phagocytes was determined on the basis of the degranulation mediated increase in the surface expression of CD11b, which is a part of complement receptor 3 primarily stored both in specific and gelatinase granules, and CD66b, primarily stored in specific granules [28]. The limited increase in CD66b on the surface of monocytes could be explained by the only limited expression of CD66b on monocytes [28]. However, data clearly showed the potential of all tested LPSs to induce the activation of both major blood phagocyte populations, which could have a significant pathological effect due to the increased potential of these leukocytes to produce pro-inflammatory cytokines and to extravasate into tissues and release free radicals and enzymes that damage local tissues and promote local or systemic chronic inflammation [38].

Blood phagocytes, as well as other innate immune cells, recognize LPS primarily by receptor TLR4 with the support of other proteins, including LPS-binding proteins, CD14, and MD-2. Upon LPS recognition, several intracellular signaling pathways are activated, among them MAP kinases leading to the activation of transcriptional factor NF-κB [27]. The LPS isolated from the environmental sample of water bloom was shown to activate the members of the MAP kinase family p38 and ERK1/2 kinases, as well as the phosphorylation of transcriptional factor NF-κB. These data clearly confirm the potential of water bloom LPS to be recognized by the major blood phagocyte population (PMNL) and to promote a complex pro-inflammatory response in these cells. We did not analyze the activation of signaling pathways in monocytes. However, we assume similar responses in these cells also according to the large quantities of pro-inflammatory cytokines produced after LPS stimulation in whole blood. Monocytes are recognized to be more potent producers of pro-inflammatory cytokines compared to PMNL, and we can assume that the majority of cytokines determined in whole blood are of monocytes production.

Information on the effects of cyanobacterial LPS is limited; thus, we focused on cyanobacterial LPS alone. Water blooms from fresh waters are known to be a mixture not only of different species of cyanobacteria but also of other bacterial genera, such as *Flavobacteria*, *Sphingobacteria*, *Bacteroidetes*, and *α-*, *β-,* and *γ-proteobacteria* [3,39]. Many of these are gram-negative bacteria containing LPS that are included in LPS isolates prepared from complex water bloom environmental samples. Therefore, the overall biological effect of such samples is a combination of the dominant effects of cyanobacteria LPS and effects of LPS of other bacteria present in that particular water bloom. To describe the biological effects of LPS from the non-axenically grown culture in contrast to those from LPS purely originating from *Microcystis*, we employed both non-axenically grown and axenic cultures of *M. aeruginosa* PCC 7806 and HAMBI/UHCC 130. Interestingly, LPS isolated from both non-axenic and axenic *Microcystis* strains induced the significant release of pro-inflammatory cytokines TNF-α, IL-8, IL-6, and IL-11β. This is in accordance with results published in the literature in which a similar response was elicited by LPS from the non-axenic strains *M. aeruginosa* PCC 7806, UTEX 2667, and A017 in whole blood [32] and the non-axenic strain *M. aeruginosa* NIES-44 in THP-1 monocytes [36]. Moreover, the pro-inflammatory effects of non-axenic *M. aeruginosa* UTCC 299 LPS, specifically the induction of IL-6 and TNFα, were reported when testing rat microglia [34]. Surprisingly, the biological effect of LPS isolated from the non-axenic *M. aeruginosa* strain was lower than the effect of LPS isolated from the axenic *M. aeruginosa* strain. In general, the activity of LPS is closely related to its structure [11], whereas some LPS were shown to be only weak agonists of TLR4 or even to be TLR4 antagonists [19,40,41]. Further, the structure of LPS is not only species and/or strain-specific but also depends on the cultivation conditions. Changes in the structure of LPS in different bacteria associated with changes in temperature and osmolarity have been described [42,43]. However, any information about the structure of cyanobacterial LPS in relation to cultivation conditions is not available in the current literature. Therefore, we cannot exclude the possibility that sterile cultivation without aeration followed by the slower growth of cyanobacteria and the lack of interactions with heterotrophic bacteria could result in axenic cyanobacterial LPS having a different—in our case, more immunotoxic—chemical structure.

LPS is also known as a pyrogen, a fever-inducing compound, and the pyrogenicity can be tested by specific enzyme-based assays. Surprisingly, our results show that the water bloom LPS had almost twice higher pyrogenic activity than *E. coli* LPS. Despite the fact that the used water bloom was dominated by *M. aeruginosa*, it also contained many different heterotrophic bacteria, among them at least one gram-negative species (data not shown). In general, the pyrogenicity of bacteria living in fresh waters can have a similar or higher pyrogenicity compared to *E. coli* [36,44]. The environmental mixture may, therefore, exert higher pyrogenicity than individual species. On the other hand, the non-axenic culture of *M. aeruginosa* PCC 7806 had more than a one hundred times stronger effect in the PyroGene test than the axenic culture *M. aeruginosa* PCC 7806. Moreover, it was more than four hundred times stronger than the *M. aeruginosa* HAMBI/UHCC 130 strain. These results are inconsistent with the fact that both axenic cultures were stronger in the activation of blood phagocytes. Similarly, the higher pyrogenicity of water bloom LPS in comparison with *E. coli* LPS does not correlate with its lower ability to activate PMNLs and monocytes. Although we do not have a clear explanation for this phenomenon, it is in agreement with observations of Dehus et al. who observed that the pyrogenicity of different LPS obtained by the *Limulus* amebocyte lysate (LAL) test does not always correlate with the activation of blood cells [45]. Apparently, interactions of cyanobacterial LPS with innate immune cells might be too complex to be predicted from routinely used pyrogenic tests, which may not capture the full range of possible biological effects.

## 4. Conclusions

This study documented the potential of LPS from non-axenic and axenic *M. aeruginosa* cultures and LPS from a water bloom sample to induce phagocyte activation manifested by degranulation and the production of a wide range of inflammatory mediators. LPS isolated from complex water bloom revealed a higher potency to activate phagocytes compared to LPS isolated from *M. aeruginosa.* However, despite this fact, our results show, probably for the first time, that LPS of axenic *Microcystis* cultures alone contributed to the overall toxicity of the complex environmental mixture and that the health risks associated with this LPS should not be underestimated.

## 5. Materials and Methods

### 5.1. Cyanobacterial Biomass Preparation

LPS fractions were extracted from a complex water bloom sample dominated by *M. aeruginosa* and from three laboratory cultures, non-axenic as well as axenic. The non-axenic strain *M. aeruginosa* PCC 7806 (a source of LPS labeled throughout the text as MA) (Pasteur Culture Collection of Cyanobacteria) and axenic (heterotrophic bacteria-free) strains *M. aeruginosa* PCC 7806 (a source of LPS labeled throughout the text as MA-A1) (Pasteur Culture Collection of Cyanobacteria) and *M. aeruginosa* HAMBI/UHCC130 (a source of LPS labeled throughout the text as MA-A2) (HAMBI/ University of Helsinki Cyanobacterial Collection) were used.

Bacterial cultures were grown in a mixture of Zehnder medium, Bristol (modified Bold) medium, and distilled water in the ratio 1:1:2 (*v*/*v*/*v*) under continuous illumination (cool white fluorescent tubes, 3000 lux) at 22 °C ± 2 °C. The *M. aeruginosa* non-axenic culture was aerated with sterilized air passed through a 0.22 µm filter [46]. In order to maintain the axenic conditions of the two axenic strains, the cultures were handled in a strictly aseptic manner and grown without aeration.

To confirm their axenic status, the cultures were periodically checked for the presence of heterotrophic bacteria. R2A agar (Merck Sigma-Aldrich, Darmstadt, Germany), which was developed for cultivation of slowly growing heterotrophic bacteria [47], was dissolved in water (1.8%, *w*/*v*), autoclaved, and poured onto sterile 100 mm Petri dishes. After solidifying, the plates were inoculated with cyanobacterial cultures (0.1 mL per plate) using the spread-plating technique and cultured at 30°C in the dark for 5–7 days prior to photo documentation. In addition, the samples of cyanobacterial cultures were examined by epifluorescence microscopy. The samples were stained with 5 µg/mL Hoechst 33342 (Thermo Fisher Scientific, Waltham, MA, USA) for 30 min and observed using 40× objective and 1.6× optovar under Zeiss Axio Observer Z1 microscope coupled with Axiocam 503 mono camera. Hoechst-stained heterotrophic bacteria were distinguished from cyanobacterial nuclei by the absence of chlorophyll fluorescence. Similarly, the cyanobacteria and heterotrophic bacteria were analyzed based on SYBR Green I fluorescence and chlorophyll *a* fluorescence using the Apogee 50 Micro flow-cytometer (Apogee, Hertfordshire, UK). Prior to this analysis, cyanobacterial cultures were stained for 15 min in the dark with 1:5000 diluted SYBR Green I original staining solution (Thermo Fisher Scientific; Cat. No. S7567).

Biomass of an environmentally relevant sample of cyanobacterial water bloom was collected from the Nove Mlyny I water reservoir on 14 August 2012 (Table 2). Cyanobacterial biomass for LPS isolation (labeled throughout the text as WB) was collected with a plankton net (20 µm mesh), transported on ice to the laboratory, and stored frozen (−20 °C) prior to lyophilization. The reservoir Nove Mlyny I is the upper reservoir of the three-reservoir cascade built on Dyje River primarily for the purposes of recreation (water sports, fishing), flood control, and hydrological management. The Nove Mlyny I reservoir is shallow (depth at the dam 3.9 m at the full reservoir level) hypertrophic-eutrophic lake with total volume 14.313 × 10^6^ m^3^ that belongs to water bodies in the Czech Republic regularly contaminated with cyanobacterial blooms dominated by *M. aeruginosa* [48]. As reported previously [49], phytoplankton cell density at the sampling site ranged between 0.135–10.2 × 10^6^ cell/mL during July–Sep 2012, peaking 1.02 × 10^7^ cell/mL in the middle of August (Table 2). During July–September, phytoplankton community was dominated by cyanobacteria (>97% of the total cell count), with specifically *M. aeruginosa* becoming nearly completely dominant (100% of cyanobacterial cell count) from the late July [49]. Overall, *Microcystis* sp. represents the most prevalent bloom-forming cyanobacterium in hypertrophic/eutrophic Czech water bodies, with typical concentrations of *Microcystis* and microcystins similar to those observed at the sampling site in this study [48,50,51,52,53]. Cyanobacterial biomass was collected with a plankton net (20 mm mesh), transported on ice to the laboratory, and stored frozen (−20°C) prior to lyophilization.

### 5.2. LPS Preparation

All cyanobacterial LPS were obtained using hot phenol-water [32,44]. A suspension of lyophilized biomass (1 g) in MilliQ water (50 mL) was sonicated, then mixed with 90% phenol (50 mL) and stirred at 68 °C for 20 min. After cooling to 4 °C, the mixture was centrifuged (5630 g/30 min/4 °C), and the supernatant (aqueous phase with LPS) was separated. The phenol layer was re-extracted with MilliQ water (50 mL). Pooled supernatants were purified by dialysis using cellulose membranes (33 × 21 mm, Merck Sigma-Aldrich, Darmstadt, Germany) against MilliQ water (1 l) containing toluene (10 μL/L) for 48 h to avoid bacterial contamination (water was changed after 24 h). Dialyzed extracts were centrifuged (5630 g/30 min/4 °C), and the supernatants were lyophilized. The semi-purified freeze-dried extract of LPS was resuspended in 3750 μL of 0.1 M Tris–HCl buffer (pH 7.4) containing 25 µg/mL ribonuclease A (Sigma–Aldrich). The solution was incubated for 16 h at 37 °C, then 3750 μL of 90% phenol in 0.1 M Tris-HCl was added. After 4 min of incubation at room temperature (RT), the solution was centrifuged (18410 g/15 min/RT), and the aqueous phase was separated and purified for 48 h by dialysis for a second time (see above) and then lyophilized. The freeze-dried powder constituting purified LPS was weighed to assess the content of LPS in the biomass and kept at −20 °C. The yield of LPS (mg/g d.w. biomass) was 7.1 +/− 3.1 (WB, *n* = 7), 13.2 +/− 6.5 (MA, *n* = 5), 12.5+/−3.2 (MA-A1, *n* = 2), 15.7 +/− 5.5 (MA-A2, *n* = 2) (average +/− SD yield from four-independent extractions). As a positive control, *Escherichia coli* LPS (serotype 026:B6) [EC], purchased from Merck Sigma-Aldrich, was used. Before experiments, all types of LPS were uniformly diluted to a stock concentration of 10 mg/mL in PBS with 0.1% BSA (Merck Sigma-Aldrich).

### 5.3. Pyrogenicity by the PyroGene^TM^ Recombinant Factor C Endpoint Fluorescent Assay

The pyrogenicity of the tested LPS samples was determined using the PyroGene^TM^ Recombinant Factor C Endpoint Fluorescent Assay (Lonza, Basel, Switzerland). The method used a one-step approach incubating 100 µL of the sample with 100 µL of the reaction solution (rFC enzyme solution, rFC assay buffer, and fluorogenic substrate in the ratio of 1:4:5). The incubation was performed in a 96-well microplate for 1 h at 37 °C according to the manufacturer’s instructions. Fluorescence was measured (excitation 390 nm, emission 440 nm) using a microplate reader, and the endotoxin activity was calculated according to the standard curve. Data were expressed as endotoxin activity in endotoxin units (EU) per mg of extracted and purified LPS.

### 5.4. Preparation of Whole Blood Samples and Experimental Layout

Blood samples were collected from healthy volunteers with given informed consent based on approval by The Ethics Committee for Research at Masaryk University (number EKV-2018-083, accessed on 5 November 2018). Blood anticoagulated with citrate was added to prevent coagulation. PMNLs were isolated as described previously [54]. The whole blood was layered in the ratio of 1:1 over a separation mixture composed of 4% Dextran-T500 (Sigma–Aldrich) and 60% Telebrix (Guerbet, Paris, France) in saline, in the ratio of 3.7:1, with a final density of 1.08 g/cm^3^. Erythrocytes were removed after 45 min of sedimentation at RT, and leukocytes with plasma were obtained. To isolate PMNLs, leukocytes with plasma were layered over Lymphoprep (Fresenius Kabi Norge, Oslo, Norway) in the ratio 1:2 and centrifuged (390 g/30 min/RT, without brake and acceleration). PMNLs were isolated, washed with RPMI 1640 (PAN; 200 g/10 min/RT), and resuspended in RPMI to a final concentration of 1 × 10^6^ cells/mL. Isolated PMNLs (3 × 10^6^ cells/mL) were used for the detection of the phosphorylation of selected kinases and NF-κB transcriptional factor by Western blot.

Whole blood samples for flow cytometry measurement were diluted 1:1 (*v*/*v*) in RPMI. The surface expression of the receptors specific for particular granules was determined after 6 h of LPS exposure. The samples of diluted whole blood (1:1) were also used for the preparation of samples for 24 h LPS exposure followed by ELISA assays to determine cytokine production. Stock solutions of all LPS samples were prepared in 0.1% BSA in PBS and added to the diluted blood or the suspension of isolated PMNLs to reach different final concentrations. In the case of the EC and WB, the final concentrations were 1, 0.1, 0.01 μg/mL. The isolated and lyophilized LPS from axenic and non-axenic *M. aeruginosa* strains were used in final concentrations of 10, 1, 0.1 μg/mL. 0.1% BSA in PBS was used as a negative control.

### 5.5. Determination of Surface Expression of Receptors by Flow Cytometry (FC)

Diluted blood was incubated with LPS at a concentration of 1 μg/mL (EC, WB) or 10 μg/mL (MA, MA-A1, MA-A2) at 37 °C for 6 h. Then, samples were incubated with rat allophycocyanin (APC) anti-human CD11b (clone M1/70) (BioLegend, San Diego, CA, USA), mouse phycoerythrin (PE) anti-human CD66b (clone G10F5), and mouse FITC anti-human CD14 (clone61D3) (both Fisher Scientific eBioscience, San Diego, CA, USA) for 15 min at RT and fixed with 4% formaldehyde in PBS for 15 min, as described previously [55]. Red blood cells were lysed using distilled water for the following 10 min at RT. After centrifugation (300 g, 7 min), the remaining cells were resuspended in cold PBS and kept on ice until the assessment of fluorescence by a FACSVerse flow cytometer (BD Biosciences, San Jose, CA, USA). Ten thousand granulocytes, selected on the basis of their typical scattering characteristics, were analyzed. Staining for the surface expression of CD14 to improve discrimination between PMNL populations (CD14^low^) and monocytes (CD14^high^) was applied. The geometric mean of the relative fluorescence for each population of PMNLs and monocytes was quantified, and final data were recalculated as a percentage of the untreated control (100%).

### 5.6. Detection of Cytokines by ELISA

Cytokine IL-1β, IL-6, IL-8, and TNF-α concentrations were determined in the supernatant obtained from diluted blood after centrifugation (5000 g/5 min/RT) by ELISA Ready-SET-Go!^®^ kits (Fisher Scientific eBioscience, San Diego, CA, USA) according to the manufacturer’s instructions.

### 5.7. Protein Analysis by Western Blot

The effects of selected LPSs on the phosphorylation of selected kinases and NF-κB transcriptional factor were determined after 15 or 30 min of the incubation of isolated PMNLs with selected LPSs at 37 °C. Then, the cells were washed in cold PBS, lysed in sodium dodecyl sulfate (SDS) buffer (50 mM Tris-HCl, pH 7.4, 100 mM NaCl, 10% glycerol, 1% SDS, 1 mM ethylenediaminetetraacetic acid, and 10^−3^ mol/l phenylmethanesulfonyl fluoride), and frozen at −80 °C. Before use, the samples were boiled and sonicated, and the concentration of proteins was determined using BCA protein assay (Pierce Biotechnology, Rockford, IL, USA), according to the manufacturer’s instructions. Cell lysates were diluted to equal protein concentrations and supplemented with Laemli buffer (200 mM Tris-HCl pH 6.8; 3% sodium dodecyl sulfate, 30% glycerol, 0.03% bromophenol blue, 3% ß-mercaptoethanol, and 200 mM dithiothreitol). 20 μg of proteins per well were then subjected to SDS-polyacrylamide gel electrophoresis, as described previously [56]. Proteins were transferred to a polyvinylidene fluoride membrane (Immobilon-P, Merck Millipore, Darmstadt, Germanyand blocked in 5% low-fat milk in TBS-T buffer (Tris, 0.05% Tween20). The membranes were incubated with primary rabbit antibodies against extracellular signal-regulated kinase (ERK1/2), phospho-ERK1/2 (Thr202/Tyr204), p38 mitogen-activated protein kinase (MAPK), phospho-p38 MAPK (Thr180/Tyr182), nuclear factor-κB (NF-κB) p65, phospho-NF-κB p65 (Ser536) (all Cell Signaling Technology, Danvers, MA, USA), and primary mouse antibody against vinculin (Merck Sigma-Aldrich) diluted in blocking buffer 5% low-fat milk in TBS-T (anti-ERK1/2; anti-p38 MAPK; and anti-NF-κB—dilution 1:2500, phospho-ERK1/2, phospho-p38 MAPK, phospho-NF-κB p65—dilution 1:1000, anti-vinculin 1:5000) on roller at 4 °C overnight. The membranes were washed extensively in TBS-T buffer 3 times at room temperature for 10 min and incubated with corresponding horseradish peroxidase-conjugated secondary antibody (Cell Signaling Technology) diluted in 1:3000 at room temperature for 1 h. The membranes were washed extensively in TBS-T buffer 3 times at room temperature for 10 min and were visualized using SuperSignal West Pico or Femto Chemiluminescent Substrate (Pierce Biotechnology) and CP-B X-ray films (Agfa). The equal loading of proteins was verified by vinculin immunoblotting. The relative levels of proteins were quantified by scanning densitometry using the ImageJ program (U. S. National Institutes of Health [57]) with the individual band density value expressed in arbitrary units (optical density, OD). The data in graphs represent mean +/− standard error of the mean (SEM) of the optical density of the phosphorylated form of the protein.

### 5.8. Statistical Analysis of Data

Data are reported as means ± SEM, and the number of independent replicates is indicated in each figure. Data were analyzed using T-test or one-way ANOVA followed by Dunnett’s test (GraphPad Prism software, version 6.0, Science Plus Group bv, Groningen Netherlands, 2012). A *p*-value (*) ≤0.05 was considered significant.

## Figures and Tables

**Table 1:** The pyrogenic activity of tested lipopolysaccharide (LPS) samples assessed by PyroGene test. Data are expressed as endotoxin activity (EU) per mg of purified LPS (mean of two independent determinations).

LPS Sample	Label	PyroGene (×10^4^ EU/mg LPS)
*E. coli* (serotype O26:B6)	EC	674
Environmental water bloom sample	WB	1210
Non-axenic *M. aeruginosa* PCC 7806	MA	108
Axenic *M. aeruginosa* PCC 7806	MA-A1	<1
Axenic *M. aeruginosa* HAMBI/UHCC 130	MA-A2	<1

**Table 2:** Characterization of water bloom biomass collected for lipopolysaccharide (LPS) isolation (sample “WB”). Adapted from Javurek et al. 2015 [49].

Locality	Nové Mlýny I, Czech Republic
Sampling Site	48°53′9.155″N, 16°35′41.663″E
Sampling Date	14 August 2012
Total phytoplankton concentration (cell/mL)	10.2 × 10^6^
Percentage of cyanobacteria in total biomass (% of the total cell count)	99.8
Species composition (% of cyanobacterial cell count)	*M. aeruginosa* (100)
The concentration of major microcystins (MCs) in phytoplankton biomass (µg/g d.w.)	MC-RR: 329 MC-YR: 83 MC-LR: 373 Sum MCs: 785
The concentration of major MCs in water (µg/L)	MC-RR: 2.22 MC-YR: 0.68 MC-LR: 1.29 Sum MCs: 4.19
